# C1q monogenic lupus: a case series and review

**DOI:** 10.1093/rap/rkaf064

**Published:** 2025-05-28

**Authors:** Israrul Haque, Kaustav Mitra, Geetabali Sircar, Parasar Ghosh, Sumantro Mondol, Subhankar Haldar, DipendraNath Ghosh, Rashmi Roongta

**Affiliations:** Department of Clinical Immunology and Rheumatology, Institute of Postgraduate Medical Education and Research (IPGMER), Kolkata, India; Department of Clinical Immunology and Rheumatology, Institute of Postgraduate Medical Education and Research (IPGMER), Kolkata, India; Department of Clinical Immunology and Rheumatology, Institute of Postgraduate Medical Education and Research (IPGMER), Kolkata, India; Department of Clinical Immunology and Rheumatology, Institute of Postgraduate Medical Education and Research (IPGMER), Kolkata, India; Department of Clinical Immunology and Rheumatology, Institute of Postgraduate Medical Education and Research (IPGMER), Kolkata, India; Department of Clinical Immunology and Rheumatology, Institute of Postgraduate Medical Education and Research (IPGMER), Kolkata, India; Department of Clinical Immunology and Rheumatology, Institute of Postgraduate Medical Education and Research (IPGMER), Kolkata, India; Department of Clinical Immunology and Rheumatology, Institute of Postgraduate Medical Education and Research (IPGMER), Kolkata, India

**Keywords:** paediatric rheumatology, systemic lupus erythematosus, rare disease, infection, genetics

## Abstract

**Objectives:**

Monogenic systemic lupus erythematosus (SLE) is caused by a single gene mutation. C1q deficiency is a rare but well-documented form of monogenic SLE, characterized by unique clinical and laboratory indicators that guide diagnosis and treatment. We aimed to describe four cases of C1q monogenic lupus.

**Methods:**

This retrospective, single-centre observational study reviews the clinical and serological profiles and outcomes of four cases of C1q Monogenic Lupus diagnosed at our centre. The study was approved by the Institutional Ethics Committee at the Institute of Postgraduate Medical Education and Research (IPGMER), Kolkata-700020 (Memo No. IPGME&R/IEC/2025/0020).

**Results:**

We describe four cases of C1Q monogenic lupus identified by whole exome sequencing. All patients exhibited mucocutaneous involvement, discoid lupus erythematosus, inflammatory polyarthritis, normal serum complements C3 and C4, coarse-speckled Antinuclear antibody positivity and antibodies to ribonucleoprotein. Unique features identified include brain parenchymal calcification in one case, chronic subdural haemorrhage in two cases, infection complicated by macrophage activation syndrome in two cases and myositis in one case. Patients were treated with conventional immunosuppressive therapy (glucocorticoids, mycophenolate, cyclophosphamide) and Fresh Frozen Plasma. Our findings were compared with existing literature on C1q deficiency, noting frequent presentations with mucocutaneous and musculoskeletal manifestations, normal C3 and C4 levels and absence of anti-dsDNA antibodies.

**Conclusion:**

C1Q Monogenic SLE should be suspected in juvenile SLE patients presenting at under 10 years, with a family history of consanguinity, predominant mucocutaneous manifestations, a history of recurrent infection, normal serum complements and absence of C1q staining in direct immunofluorescence of renal biopsy. In our series, autoimmune manifestations responded well to immunosuppressive therapy.

Key messages

- Consider monogenic C1Q deficiency in early-onset SLE with preserved complement levels.
- Whole exome sequencing confirms the diagnosis when clinical and serological features are suggestive.
- Autoimmune manifestations are typically responsive to immunosuppression; however, infection remains a significant clinical threat.

## Introduction

Systemic lupus erythematosus (SLE) is an autoimmune disease predominantly affecting females (M:F ratio 1:9), with early-onset forms carrying a poorer prognosis. Rare monogenic forms of SLE, caused by mutations in genes like C1q, C1r, C1s, C4, C2, C3, TREX-1, DNase I, DNase IL3, AGS5 and ACP5, display distinct clinical patterns [1]. The 2019 EULAR/ACR classification criteria represent the latest, highly sensitive diagnostic guidelines [2].

## Materials and methods

This retrospective, single-centre observational study reviews the clinical and serological profiles and outcomes of four cases of C1q Monogenic Lupus diagnosed at our centre. The study was approved by the Institutional Ethics Committee at the Institute of Postgraduate Medical Education and Research (IPGMER), Kolkata-700020 (Memo No. IPGME&R/IEC/2025/0020). Informed consent was taken from parents of 3 patients.

All cases were diagnosed using Whole exome sequencing. Sequencing of ∼30 Mb of the human exome (covering ∼99% of CCDS and RefSeq regions) and the complete mitochondrial genome was performed using Illumina NGS platforms. Mean sequencing depth was 80–100× for exome and 1000–2000× for mitochondrial genome, with >90% of exome bases covered at ≥20×. Exons were considered fully covered if all coding bases plus ±3 flanking nucleotides had ≥20× depth. Variant calling followed GATK best practices and was processed on the DRAGEN BIO-IT platform, including duplicate removal, base recalibration and indel realignment. VCF quality checks included variant type distributions, common variant ratios and extreme heterozygote identification. Variants were annotated using OMIM, GWAS, gnomAD and 1000 Genomes databases. Only non-synonymous and splice site variants were interpreted clinically. Annotation and reporting were done via Geneyx software (v5.12). Pathogenicity in whole exome sequencing is assessed using tools like REVEL, which integrates multiple predictive algorithms. High REVEL scores (closer to 1), rarity in population databases, conserved residue impact and ClinVar classification support pathogenicity.

Case 1: A 5-year-old girl with consanguinity presented at age 2 with myocarditis, rash and arthritis. Whole exome sequencing (WES) revealed a *C1QA* mutation. Despite normal coagulation profile, she developed subdural haemorrhage. She developed pyogenic meningitis and herpes zoster during follow-up and died of probable sepsis after being lost to follow-up.

**Figure 1.**
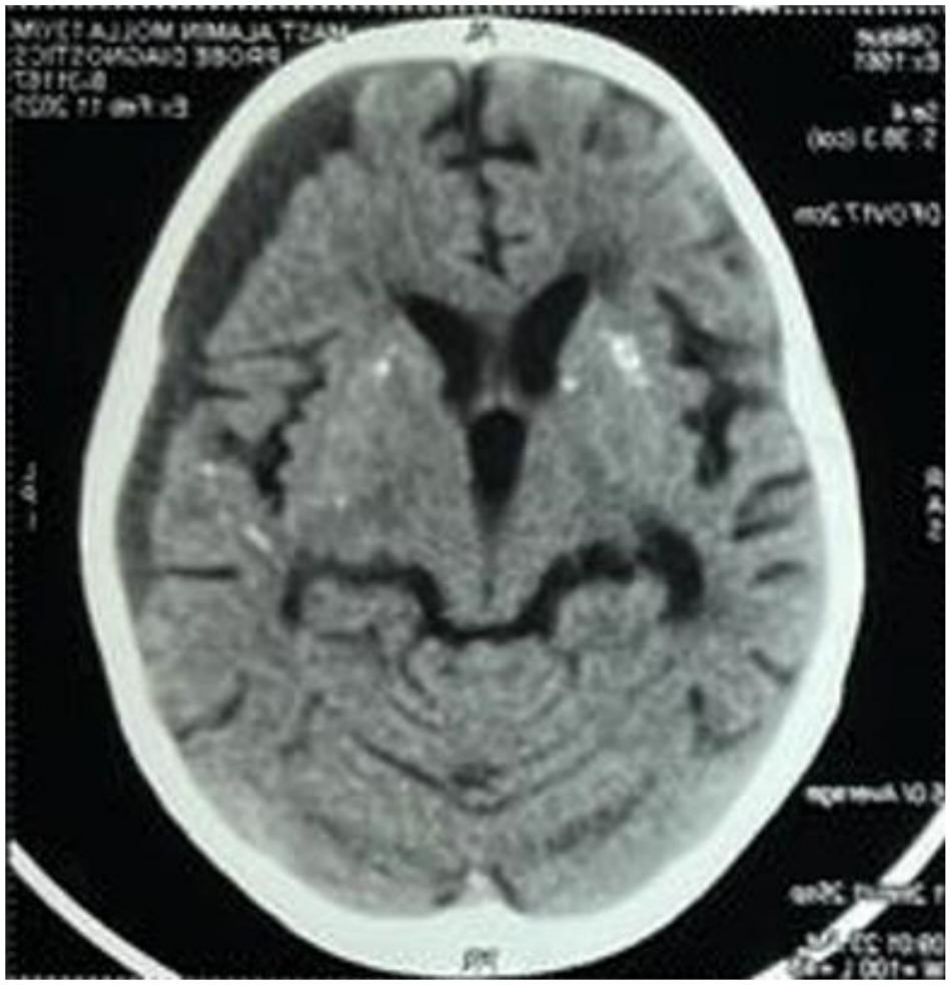
MRI brain of patient 2

Case 2: A boy diagnosed at 13, with SLE symptoms since age 6, had chronic subdural haemorrhage and basal ganglia calcifications (Fig. 1). WES showed a likely pathogenic exon 2 *C1QA* mutation. He developed macrophage activation syndrome (MAS) from a staphylococcal abscess which was Managed with intravenous immunoglobulin (IVIG), corticosteroids, and currently he is in DORIS remission.

Case 3: A girl diagnosed at 10 years of age with biopsy-proven mesangioproliferative glomerulonephritis (GN) lacked C1Q/C3 on direct immunofluorescence (DIF). WES revealed autosomal recessive mutations in *C1QA* and *INPP5E*. Treated as per NIH protocol and transitioned to mycophenolate mofetil, she remains in remission.

Case 4: A boy with recurrent infections since age 2, diagnosed with SLE at 6, had a novel homozygous *C1QB* mutation. He experienced a seizure and MAS secondary to streptococcal pneumonia. Managed with tacrolimus, IVIG and corticosteroids; he remains in remission.

ACLE presented as transient malar erythema sparing nasolabial folds; SCLE as annular or psoriasiform erythematous, non-scarring photosensitive plaques over upper limb, upper back and neck; and DLE as scaly, indurated plaques with follicular plugging and post-inflammatory hypopigmentation. All diagnoses were made clinically and confirmed by the dermatology team in each case. Serum complement C3 and C4 levels were within normal range in all patients throughout follow-up. All patient details are summarized in Table 1.

**Table 1.** Summary of clinical, immunological and important laboratory features of cases

Features	Case 1	Case 2	Case 3	Case 4
Age at last follow up, years	5.5	13	10	18
Sex	Female	Male	Female	Male
Ethnicity	South Asian	South Asian	South Asian	South Asian
Age of onset of symptoms, years	2	6	2	2
Age of diagnosis, years	5	12	10	6
Method used for diagnosis	Whole exome sequencing (WES)	WES	WES	WES
Gene mutation identified	C1QA	C1QA	C1QA INPP5E^••^	C1QB
Details of mutation	Homozygous mutation	c.44delT in Exon 2 Homozygous mutation, Autosomal recessive (AR)	Homozygous c.369delC in exon 3 of C1QA, AR Homozygous c.2_10delTGCCGTCCA p.Met1_ser3del mutation in exon 1 of INPP5E, AR	c.668 A > C in exon 3 homozygous mutation, AR
Significance of mutation	Undetermined significance^a^	Likely pathogenic (novel mutation)	Likely pathogenic mutation in both C1QA gene and INPP5E gene.	Uncertain significance (novel mutation)
Impact of mutation on the protein^#^	NA	High	high on C1QA protein and medium on INPP5e protein	Medium
H/o consanguinity	Yes Second generation	Yes Second generation	No	No
history of unexplained death/SLE in sibling	Yes, Unexplained death of sibling at years of age 4/no	No/no	No/no	No/no #
Satisfies ACR 1997 classification criteria for SLE	Yes	Yes	yes	Yes
2019 Eular/ACR classification criteria^2^ for SLE Score	22	28	22	24
Clinical features (Ever)				
Constitutional	Fever Weight loss	Fever Weight loss	Fever	Fever
Mucocutaneous	Skin rash with pustules, Post-inflammatory hyperpigmentation, DLE, Alopecia, Palatal ulcer, Photosensitivity	Photosensitivity Malar rash, SCLE rash, DLE, Livedo reticularis, Palatal ulcer, Post-inflammatory hyperpigmentation.	ACLE rash, DLE rash, Alopecia, Photosensitivity	Palatal ulcers Malar rash DLE Vasculitis rash
Musculoskeletal	Inflammatory polyarthritis (IPA)	IPA, Myositis (weakness in legs≫arm) leading to flexion deformity of knees	IPA	IPA
Renal	Absent	Absent	mesangioproliferative nephritis	24 h urine protein 1263 mg
CNS^##^	5.1× 1.8 cm Chronic subdural haemorrhage in left fronto-parietal area, chronic maxillary sinusitis, Focal seizure	Multifocal bilateral Intracranial calcification involving basal ganglia and cerebral hemisphere, Chronic Subdural haemorrhage involving right temporo-parietal area.	Subnormal intelligence	One episode of GTCS, Transient bilateral painless dimness of vision for 1 days (aetiology?)
Haematological	Anaemia of chronic disease (ACD)	Leukopenia	Leukopenia	ACD
Cardiovascular	Myocarditis	Normal	Normal	Normal
Gastrointestinal	Absent	Absent	Absent	Absent
Genitourinary	Absent	Absent	Absent	Absent
History of MAS	No	Yes	No	Yes
Immunological				
ANA	4+	3+	2+	3+
Pattern	Coarse speckled	Coarse speckled	Coarse speckled	Coarse speckled
ANA immunoblot	RNP/Sm+++ SSA +++ Ro52+++	RNP/Sm++ Sm+	RNP++	RNP/Sm++ SSA 3+ SSB 3+
Any other autoantibody	No	Ku+(low titre)	No	No
C3/C4, mg/dl (90–180)/(10–40)	105/65	122/34	119/47	122/31
dsDNA, IU/ml (<100 IU/ml)	<10	108	6.67	<10
1 or more antiphospholipid antibody positivity	Absent	Absent	Absent	Absent
Serum total IgG/IgA/IgM (mg/dl)	1250/275/347	1560/253/317	1580/198/280	1260/275/390
Haemoglobin, g/dl (13–17)	8.9	11.2	12.7	10.8
MCV, FL (83–101)	69	74	78	88
Total leucocyte count, 10^3^/µl (4–10)	7.3	3.3	3.4	4.8
Platelet, 10^3^/µl (160–140)	530	250	150	220
Urea, mg/dl (21–43)	13	18	16	16
Creatinine, mg/dl (0.8–1.5)	0.2	0.4	0.4	0.6
SGPT/SGOT (U/l) (10–50/17–59)	23/18	58/68	33/62	22/19
Creatinine phosphokinase (U/l) (29–168)	NA	53	32	54
Urine routine	NAD	NAD	Protein+, RBC-3-4/Hpf, WBC 3–4 Hpf	Protein+, RBC 2-3/Hpf, WBC 0-1/Hpf
Baseline 24 h urine protein, mg/24 h	110	111	1800	1283
Renal biopsy	Not done	Not done	Mesangioproliferative Glomerulonephritis C3, C1Q negative in DIF	Not done
SLEDAI-2K score at diagnosis of SLE	11	23	19	16
Therapy	Prednisolone Hydroxychloroquine (5 mg/kg) Azathioprine (1–2 mg/kg) FFP	Prednisolone Hydroxychloroquine (5 mg/kg) Methotrexate (10 mg/m^2^) (IVIG), FFP	Prednisolone Hydroxychloroquine (5 mg/kg) CYC*** 500 mg/m^2^ 6 doses then Mycophenolate mofetil 1 g/day	Prednisolone Hydroxychloroquine (5 mg/kg) Tacrolimus 1 mg twice daily IVIG
SLEDAI score at 6 month after therapy	0	0	0	0
History of serious infections	Yes Pyogenic meningitis	Yes Pre-patellar abscess	No	Yes Lobar Pneumonia
Microbiological diagnosis of serious infection	NA	Staphylococcus aureus	Not relevant	Streptococcus pneumonia
Any other genetic condition	B-thalassemia trait	No	No	no
Any other autoimmune disease	No	No	No	Autoimmune thyroiditis
Current status (alive/expired)	Expired	Alive	Alive	Alive
Cause of mortality	Sepsis	–	–	–

a The details of mutation were not shared by the reference institution.

## Discussion

Monogenic SLE refers to rare, inherited forms of SLE resulting from mutations in a single gene. Mutations in the C1Q gene impair the classical complement pathway, essential for clearing apoptotic cells and immune complexes which predisposes to autoimmunity [1]. Clinical manifestations in C1Q deficiency include SLE-like features, history of recurrent infection or may be asymptomatic at diagnosis [3, 4]. Stegert *et al.* [3] reported that 55% of 71 patients met ACR SLE criteria, while 22.5% had SLE-like features. In this manuscript, C1Q monogenic SLE is defined as fulfilling the 2019 EULAR/ACR SLE criteria along with detection of a C1Q mutation via whole exome sequencing [3]. All four patients in our case series met ACR-1997, SLICC-2012 and EULAR/ACR-2019 criteria [2].

Compared with multifactorial juvenile SLE, C1Q monogenic SLE typically presents earlier (median <5 years) [3], often in consanguineous families, with equal sex distribution. Features such as discoid rash, oral ulcers, anti-Smith antibodies and recurrent infections are more frequent in C1Q lupus [3, 4, 5]. Renal and neurological involvement are similar to juvenile SLE, but arthritis, ANA positivity and anti-dsDNA antibodies are less common in C1Q monogenic lupus [3].

Our series includes four South Asian patients: three with C1QA mutations, one with a C1QB mutation. The female:male ratio was 1:1; median symptom onset was 2 years. All exhibited mucocutaneous involvement, DLE rash, polyarthritis, coarse-speckled ANA, anti-RNP antibodies and normal C3/C4 levels. Notably, only one patient had anti-dsDNA antibodies. Two had nephritis of which one had biopsy-proven mesangioproliferative GN with absent C1q and C3 on DIF. Similar histopathological patterns have been previously reported [3, 5, 6]. Although hypocomplementemia is common in C1Q lupus, all our cases had normal complement levels, in contrast to Al-Mayouf *et al.* [5], who reported hypocomplementemia in 84.6% of patients. None of the cases had family history of SLE; however, two cases involved second-degree consanguinity, and one had a sibling who died unexpectedly.

Unusual manifestations in our series include chronic subdural haemorrhage in two patients, despite normal coagulation. While ischaemic cerebral infarction is known in C1Q lupus, subdural haemorrhage is not previously reported [7]. One child had myositis with flexion contracture of knees, a novel finding in this context. Another had basal ganglia and hemispheric calcifications, aligning with findings by Al-Mayouf *et al.* [5].

Three patients had recurrent infections; one likely died of infection after follow-up loss. Two developed infection-associated MAS**—**one post-staphylococcal abscess, another post-lobar pneumonia. Both responded well to methylprednisolone, IVIG and antibiotics. While MAS occurs in 0.9–4.6% of SLE cases, reports in C1Q monogenic SLE are lacking [8]. A comparative chart summarizing the clinical and serological features of our C1Q monogenic SLE series alongside published cohorts is provided in Supplementary Table S1, available at *Rheumatology Advances in Practice* online.

Diagnosis in all cases was confirmed via whole exome sequencing. Mutations included three in C1QA and one in C1QB, all homozygous. Two were novel. Due to financial and resource limitations, CH50 and serum C1Q levels were not assessed. We considered mutations of undetermined significance as causative due to the rarity of C1Q lupus, absence of variant databases and alignment of clinical features with reported cases [5, 6, 9].

Treatment focused on controlling autoimmune activity using steroids, methotrexate, azathioprine, cyclophosphamide, mycophenolate and tacrolimus, as per disease severity. IVIG and fresh frozen plasma (FFP) were used during infections. One patient was scheduled for prophylactic FFP (15 mg/kg every 3 weeks) but was lost to follow-up. FFP infusions have shown improvement in some reports [10]. Prophylactic antibiotics and meningococcal vaccination may reduce infection risk in these patients [6]. Though haematopoietic stem cell transplant (HSCT) has shown success in refractory cases [6], it carries significant procedural risks. Arkwright *et al.* [11] reported normalization of serum C1q after HSCT for severe disease.

At baseline, the median SLEDAI score was 17.5. Three patients are currently in DORIS remission, achieved within 6 months of therapy. Two have damage—one having hypopigmentation from DLE and another having knee contractures from myositis. Al-Mayouf *et al.* [5] reported growth failure and cutaneous damage in their cohort. Unlike their study, where SLEDAI scores remained high despite therapy, our cases showed favourable response to treatment.

Defects in the immune system can lead to both uncontrolled infections and autoimmune responses. Examples include Familial Hemophagocytic Lymphohistiocytosis (FHL), Chronic Granulomatous Disease AND Wiskott–Aldrich syndrome. These cases underscore the dual nature of immune system malfunctions, affecting both the body's defence against pathogens and its self-tolerance [12].

Limitations of our study include small sample size and lack of C1Q protein quantification. However, strengths include detailed longitudinal data and description of previously unreported features—subdural haemorrhage and MAS.

In summary, C1Q monogenic SLE presents early in life, with both common and rare manifestations, and may lack typical serologic markers. Despite recurrent infections and severe presentations, outcomes are favourable with timely immunosuppressive and supportive therapy.

## Supplementary Material

rkaf064_Supplementary_Data

## Data Availability

No new data has been created. All relevant clinical and genetic details have been provided within the manuscript and in the supplementary table, available at *Rheumatology Advances in Practice* online.
