# Supplementary material for: C1q monogenic lupus: a case series and review
Source: Rheumatol Adv Pract. 2025 May 28;9(3):rkaf064. doi: 10.1093/rap/rkaf064 (PMC12187074; doi:10.1093/rap/rkaf064)
Supplement: rkaf064_Supplementary_Data [file rkaf064_supplementary_data.pdf]

Supplementary Table 1: Comparison of clinical and immunological features of our series with other available literature on monogenic lupus due to c1q mutation

	Our series (n=4), N (%)	M.Stegert et al. (n=55) [3] N (%)	Al-Mayouf et al. (n=26) [5] N(%)	Olsson et al. (n=5) [6] N (%)
Satisfying ACR 1997 classification criteria	4 (100)	55(100)		4(80%)
Satisfying SLICC criteria	4(100)		**34/39(87.17)	
History of consanguinity	2 (50)	24(44.44)	15(57.6)	NA
Family history of SLE	0	NA		4(80)
Median SLEDAI at Diagnosis	17.5	NA	6	NA
Median age at symptom onset, years,	2	5	3.9	1.25
Gene mutation identified	C1Q (3 C1QA, 1 C1QB)	NA	C1Q	All 4 C1QA
female: male	1:1	1.1:1	1.6:1	1:4
History of infection	3(75)	22(40)	22(84.6)	2(40)
Mucocutaneous involvement	4		25(96.2)	
Acute cutaneous Lupus erythematosus*	4(100)	31(56.4)	71.8%#	3(60)
DLE*	4(100)	31(56.4)	56.4%#	1(20)
Photosensitivity	4(100)	25(45.5)	NA	NA
Inflammatory polyarthritis	4(100)		71.8#	NA
Myositis	1(25)	NA	NA	NA
Serositis*	0	5 (9)	12.8%#	NA
Nephritis*	2(50)	17(31)	9(34.6)	1(20)
Neuropsychiatric*	2(50)	13(23.6)	5(19.2)	1(20)
Anaemia (AOCD/AIHA)	2(50)	3(5.4)	46.2%(all AIHA)#	1(20)
Leukopenia and/or lymphopenia*	2(50)	9(16.3)	87.2#	1(20)
Raised dsDNA (>100 IU/mL)	1(25)	10(18.2)	NA	NA
Decreased C3 (<90 mg/dL)	0	NA	84.6%##	NA
Decreased C4 (<10 mg/dL)	0	NA		NA
Positive ANA	4(100)	49(89)	100%#	3(60)
Most common ANA pattern	Coase specked (100%)		NA	Speckled (2/3)
Most common Autoantibody	RNP/Sm 4(100)	NA	NA	NA
Smith	2(50)	38.8%	NA	NA
1 or more APLA	0	NA	NA	NA
Macrophage activation syndrome	2(50)	NA	NA	NA
Immunosuppressants used	Corticosteroids<br>IVIG, HCQ<br>Methotrexate<br>Cyclophosphamide<br>Methotrexate<br>Azathioprine, FFP	NA	Corticosteroids<br>Cyclophosphamide<br>Mycophenolate Mofetil<br>Rituximab<br>Belimumab<br>HCQ, IVIG	Prednisolone<br>HCQ<br>Azathioprine<br>Rituximab<br>FFP
Mortality in follow up	1(25)	NA	4(15.4)	NA
Most common Cause of mortality	Sepsis		Serious Infection	NA

RNP/Sm: Ribonucleoprotein/smith, * as per the definition given in 2019 EULAR/ACR classification criteria [2], NA: not available, HCQ: Hydroxychloroquine, APLA: Antiphospholipid antibody, SLICC: Systemic Lupus internation Collaborating Clinic, **The series had 39 patients of which 26 had C1Q deficiency and 34 fulfilled SLICC classification criteria, # the series has 39 patients, of which 26 were C1Q monogenic lupus patients and percentages has been reported for all 39 patients as separate data for C1Q population not available, ##: Reported in study as hypocomplementemia, AOCD: Anemia of chronic disease, FFP: fresh frozen plasma, IVIG: intravenous immunoglobulin.
